# Children's Behavioral Pain Cues: Implicit Automaticity and Control Dimensions in Observational Measures

**DOI:** 10.1155/2017/3017837

**Published:** 2017-02-21

**Authors:** Kamal Kaur Sekhon, Samantha R. Fashler, Judith Versloot, Spencer Lee, Kenneth D. Craig

**Affiliations:** ^1^School of Dental Medicine, Case Western Reserve University, Cleveland, OH, USA; ^2^Department of Psychology, York University, Toronto, ON, Canada; ^3^Mississauga Academy of Medicine, University of Toronto, Toronto, ON, Canada; ^4^Department of Computer Sciences, University of British Columbia, Vancouver, BC, Canada; ^5^Department of Psychology, University of British Columbia, Vancouver, BC, Canada

## Abstract

Some pain behaviors appear to be automatic, reflexive manifestations of pain, whereas others present as voluntarily controlled. This project examined whether this distinction would characterize pain cues used in observational pain measures for children aged 4–12. To develop a comprehensive list of cues, a systematic literature search of studies describing development of children's observational pain assessment tools was conducted using MEDLINE, PsycINFO, and Web of Science. Twenty-one articles satisfied the criteria. A total of 66 nonredundant pain behavior items were identified. To determine whether items would be perceived as automatic or controlled, 277 research participants rated each on multiple scales associated with the distinction. Factor analyses yielded three major factors: the “Automatic” factor included items related to facial expression, paralinguistics, and consolability; the “Controlled” factor included items related to intentional movements, verbalizations, and social actions; and the “Ambiguous” factor included items related to voluntary facial expressions. Pain behaviors in observational pain scales for children can be characterized as automatic, controlled, and ambiguous, supporting a dual-processing, neuroregulatory model of pain expression. These dimensions would be expected to influence judgments of the nature and severity of pain being experienced and the extent to which the child is attempting to control the social environment.

## 1. Introduction

Success in communicating an experience of pain to others can be a vital social transaction when there are threats to personal safety [1]. Observer skill in understanding the location, quality, and severity of pain, as well as the reason for pain expression, may facilitate delivery of care. The capacity to signal pain to others is manifest in the neonate [2, 3] and improves with the advent of language and rapidly expanding vocabularies of words related to pain in children [4, 5]. Systematic and validated self-report scales have been developed to measure pain in children [6], although they have important limitations [7]. Children may be (1) too young to understand or use the self-report scale; (2) experiencing pain that is too severe for use of self-report; (3) cognitively or communicatively impaired; (4) restricted from use of self-report by bandages or mechanical ventilation; or (5) voluntarily suppressing or exaggerating their report of pain [8, 9]. In consequence, observational pain scales focusing upon nonverbal behaviors have emerged to provide comprehensive pain assessment of children [7], either in conjunction with self-report or when self-report is not available or unreliable [10].

Judgments of pain in others represent complex decisions influenced by both characteristics of observers and available information [11], with the behavior of the person in pain a prime source. Various behaviors signaling pain cannot be construed as equivalent or conveying similar information [12]. The most typical distinction is made between verbal and nonverbal expressions, with verbal expression valued because it provides access to subjective experiences. A further distinction has been made between behaviors whose primary function is communication, for example, speech or facial expression, and behaviors whose primary function is protective, for example, postural adjustments, reflexive withdrawal, or use of medications. With protective behaviors, the signal value is secondary to the impact the action may have in avoiding, diminishing, or escaping pain [13]. A recent distinction, based on our understanding of fundamental neuroregulatory systems [14], has been made between pain behaviors that are automatic and reflexive in nature, such as nociceptive reflexes, facial expression and paralinguistic features of vocalizations, and controlled or deliberate actions, such as self-report, help seeking, and self-administering medication [15, 16]. Adults consistently make this distinction when characterizing the pain actions of others. McCrystal and colleagues [17] established that, consistent with dual neuroregulatory theory, certain adult pain behaviors were reliably characterized as automatic, others controlled, and some remained ambiguous. This categorization informs our understanding of clinical judgments of pain, since pain behaviors that are perceived to be controlled provoke greater uncertainty as to their source [15, 18, 19].

The purposes of the current study were (1) to compile a comprehensive list of all of the pain cues in all identified observational pain scales for children aged 4 to 12 years and (2) to establish whether the cues systematically varied in terms of whether they were perceived as automatic or controlled. The long-term objective is to develop a preliminary scale composed of items that reflect automatic, controlled, and ambiguous pain expression.

## 2. Materials and Method

### 2.1. Systematic Review

The systematic review was conducted according to PRISMA guidelines [20]. Online literature searches were conducted using MEDLINE (1965–January, 2011), PsycINFO (1967–January, 2011), and Web of Science (1900–January, 2011) databases. The last search was made in January, 2011. For the initial search, the study had to have been published in English. For each database, searches were made using a combination of three terms. The first term was “pain assessment”, “pain rating”, or “pain scale”. The second term was “pediatric” or “children”. The last term was “observation^*∗*^” or “behavior^*∗*^”. In Web of Science, search terms were entered in the following format: “TS = (Pain assessment AND pediatric AND behavior^*∗*^)”. In PsycINFO, search terms were entered in the format: “Pain assessment AND pediatric AND behavior^*∗*^”. PubMED searches were made using RefWorks and conducted in the following format: “Pain assessment AND pediatric AND behavior^*∗*^ AND (English[lang])”. All possible combinations were pursued using the aforementioned terms, with 16 searches conducted for each database. After deletion of duplicate articles, 1595 articles remained. The publication date of the articles ranged from 1968 to 2011.

Relevant articles were chosen according to specific inclusion criteria. First, articles were screened by title to exclude articles that did not meet the required age range (4 to 12 years). Titles containing the terms “infant,” “toddler,” “adolescent,” “adult,” and “elderly” were deleted, although titles that included the word “child” or a synonymous term were kept for further review. Articles with ambiguous titles were retained for further screening. Second, articles not published in peer-reviewed journals were excluded to maintain the empirical integrity of the included articles, eliminating commentaries, dissertations/theses, and book chapters. Review articles were retained as potential sources of relevant scales through the “snowball method” that is effective to identify obscure articles that might otherwise be overlooked [21]. In total, 418 articles were eliminated. Third, the abstracts of each remaining article were screened and only abstracts including a description of the development of an observational pain scale for children were retained. Articles that exclusively examined self-report as pain measures, for example, visual analogue scales or numerical markers, were excluded. If the abstract seemed relevant but did not mention use of a scale, the article was kept for further review. This process eliminated 1072 articles. The final step consisted of the review of the full paper. One hundred and seventeen full articles were reviewed to see if they met inclusion criteria.

A total of 21 articles that met the criteria were found in the literature search [22–44]. One article identified in a recent review by von Baeyer and Spagrud [7] was added using the snowball method [29]. The final list contained scales that included self-report items (e.g., the Princess Margaret Pain Assessment Tool [38]) and physiological items (e.g., the COMFORT scale [22]), but these were retained as long as they also contained nonverbal behavioral items. For scales that had been modified or revised over time, the scale with the greater number of behavioral cues was retained, in line with the intent to create an exhaustive list of observational pain cues in the published empirical literature. The most common revision reduced the number of scale items (e.g., the Parents' Postoperative Pain Measure was reduced from 23 indicators to 10 in the revised version [28]).

### 2.2. Compilation of Cues

The 21 articles yielded 368 cues, but these often were unclear and some represented combination items, as noted below. Compiling a list of independent items referring to pain behaviors was accomplished using the following criteria. (i) Combined/composite behaviors were separated into individual items. For example, the Noncommunicating Children's Pain Checklist-Revised [25] has the item “a change in eyes, including squinching of eyes, eyes opened wide, eyes frowning.” This was split into four separate behaviors, “a change in eyes,” “squinching of eyes,” “eyes opened wide,” and “eyes frowning.” (ii) Items that were incompatible with pain were eliminated, for example, “happy,” “neutral,” and “relaxed” (these seem to be used as they are associated with an absence of pain). (iii) Items were eliminated that did not represent acute pain or exacerbated chronic pain, for example, items related to quality of life, respiratory distress, or anxiety. (iv) Nonspecific qualifying terms were eliminated, for example, indefinite adjectives or adjectives that qualify the action affectively. For example, for the cue “negative grimace,” the word “negative” was removed and for the cue “softly crying,” the word “softly” was removed. However, important qualifiers were retained, such as the word “quietly” in the item “quietly breathing.” And (v) items designed to summarize observations were eliminated, such as a global term for judgments of the degree of pain, rather than referring to a specific action or verbal statement. According to these criteria, certain items were eliminated and others added, leading to a final total of 381 cues.

In order to eliminate redundant items, five raters independently reviewed the list of 381 items. The raters were instructed to nominate items for exclusion that were identical, closely related, or synonymous (e.g., “grimace” and “grimacing”), vague or nondescriptive (e.g., “altered expression”), and referring only to an emotion (e.g., “angry”), incorporated features of other items (e.g., “reluctant to smile” as an element of the item “inconsolable”), and only quantified the behavior in terms of time (e.g., “grimace for less than 50% of the time” and “long grimace > 50% of time”). Cues with 100% agreement for inclusion among the raters were retained. Cues with 20–80% agreement concerning retention were discussed until 100% consensus concerning inclusion or exclusion was reached.

### 2.3. Automaticity Rating Procedure

To determine the degree of automaticity of the observational pain cues, an online survey was undertaken. Participants were instructed to picture a child between the ages of 4 and 12 years who was experiencing pain and exhibiting a wide range of painful actions. The participant was instructed to rate each pain behavior cue according to their position on seven 10-point Likert subscales using anchors developed by Moors and De Houwer [45]: “goal-oriented versus nongoal-oriented,” “conscious versus unconscious,” “uncontrolled versus controlled,” “fast versus slow,” “intentional (deliberate) versus unintentional,” “stimulus-driven (obligatory) versus self-driven,” and “requiring contemplation versus not requiring contemplation.” These subscales have been used in previous research to identify automatic and controlled pain behaviors, but not with children [17].

The survey was posted on the online hosting service Mechanical Turk (http://www.mechanicalturk.com). Mechanical Turk is managed by Amazon.com and allows for the completion of simple tasks by research participants recruited from a pool of over 100,000 users in over 100 countries. Research participants characterized themselves on basic demographic scales. Users received a small monetary stipend for completing the task that could be predetermined when the survey was posted. For the present study, the stipend amount selected was $2.00, a rate higher than the standard compensation amount [46]. Advantages of using Mechanical Turk include that it provides fast and reliable recruitment and shows few differences with more standard undergraduate populations for nonresponse errors, attention, and reliability [47]. Mechanical Turk also can provide a more representative sample of the general population than a more standard sample of undergraduate students in psychology [46, 48]. Disadvantages of using Mechanical Turk include that the recruited sample is at risk of being skewed to represent a country that has a higher proportion of registered users.

The study protocol was approved by the Behavioral Research Ethics Board at the University of British Columbia. Written informed consent was obtained from participants before beginning the study.

## 3. Results

### 3.1. Children's Behavioral Pain Cues

Application of the criteria described above yielded a list of 66 cues. These items appear in Tables 1–3.

### 3.2. Survey Data Preparation

A total of 296 participants completed the online survey. The data were screened for duplicate IP addresses and for nonsensical responses (e.g., the same response on all of the Likert scale for all questions). This resulted in the exclusion of the data of 19 participants, leaving 277 complete data sets. To prepare the data, three of the automaticity subscales were reverse-scored and the average of all seven scales was calculated in line with methods utilized in previous research [17]. In this manner, one automaticity score was calculated for each pain behavior item per participant. A score of “1” represented the extreme automatic rating, while “10” represented the extreme controlled rating.

### 3.3. Participants

The majority of participants were male (65%). Participant mean age was 24 years and ranged from 18 to 65 years. All were fluent in English. The majority of participants cited English as their first language (*N* = 122), while the first language of 121 participants was a South Asian language (e.g., Hindi, Malayalam, and Tamil). The first language of the remaining participants consisted of 17 other languages. The majority of participants 76.6% (212) were born in India, with 33 participants born in the United States. A majority of participants (210) also lived in India and 39 participants lived in the United States.

### 3.4. Factor Analysis

**Figure 1:**
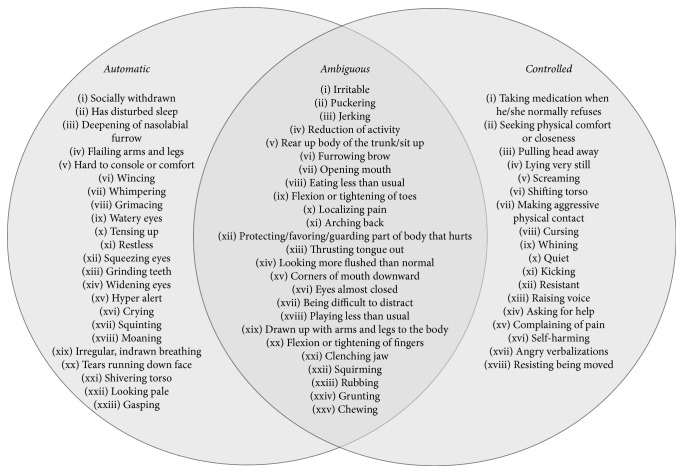
Pain behavior items categories according to the factor analysis.

To determine the factorial structure of these ratings, an Exploratory Factor Analysis was conducted using Unweighted Least Squares (ULS) extraction with a Direct Obliman [49]. Direct Obliman was considered the most suitable as it was unknown if the derived factors were correlated [50]. Items with a loading of 0.32 or higher were considered adequately loaded [51]. When a variable was loaded on two factors, a difference of at least 0.15 was needed to assign the item to the higher loaded factor [51]. It was found that 18 behaviors loaded on factor 1 (that we labeled as the “Controlled” factor), with 12 being highly loaded (above 0.60). Factor 1 accounted for 18.26% of the total explained variance explained. Twenty-three behaviors loaded on factor 2 (labeled as the “Automatic” factor), with three being highly loaded (above 0.60). Factor 2 accounted for 16.93% of the total variance explained. There were 10 behaviors on factors 3 or 4 and 14 behaviors that loaded on two or more factors, and one cue did not load on any factor and was categorized as ambiguous. All of these remaining items were labeled as “Ambiguous.” Factor 3 and factor 4 accounted for 3.69% and 3.16% of the total variance explained, respectively. See Tables 1–3 for a full list of cues and their factor loadings.  Figure 1 provides a graphic representation of the items according to their categories.

## 4. Discussion

### 4.1. Types of Cues

A considerable diversity of actions has been identified as signifying pain in children, including behaviors that could be characterized as verbal (e.g., “asking for help,” “complaining of pain,” and “cursing”), facial activity (e.g., “wincing,” “furrowed brow,” and “widening eyes”), nonverbal vocalizations (e.g., “whimpering,” “crying,” and “moaning”), limb action (e.g., “flailing arms and legs,” “rubbing,” and “protecting/favoring/guarding part of body that hurts”), body action (e.g., “tensing up” and “restless”), physiological manifestations (e.g., “looking pale,” “irregular breathing,” and “shivering torso”), and social behaviors (e.g., “withdrawn,” “hard to console,” and “angry verbalizations”). While there were commonalities across scales in the general categories employed, there was also considerable diversity in how actions in the different domains were described.

Descriptions of methods used by authors of the original papers to select items for inclusion in their scales referred primarily to expert judgment, either provided by the authors of the scales themselves or surveys of others who spend time with children in pain, for example, nurses or parents. Exceptions to personal nomination of items or the use of items already described in the literature were studies that used direct observation of children (e.g., [32, 41]). While most of the items appearing in Tables 1–3 seem to have face validity (i.e., items appear to measure what they were purported to measure), some appear problematic or lacking in item validity (i.e., whether the item discriminates children who are in pain from those who are not). Some items are contrary to other proposed items, for example, reduction of activity or quiet versus jerking, arching back, or kicking. Others are very broad, for example, having disturbed sleep, eating less than usual, being irritable, and playing less than usual. While these behaviors may arouse suspicion concerning the presence of pain, they are nonspecific and associated with a wide diversity of nonpainful conditions. Ethological investigations that provide fine-grained descriptions of behavior specific to particular populations in specific situations are required (e.g., Warnock and Sandrin [52]).

There is room for improvement in descriptions of items. Chang et al. [53] found that behavioral descriptions of the facial expression of pain are often problematic. Some items are not sufficiently detailed as to allow discrimination of pain from nonnoxious but aversive states, for example, wincing and grimacing. Items are used that are contraindicated by empirical findings as they have not been reported as associated with pain in children, for example, “puckering” and “thrusting tongue out.” The item “eyes opened wide” appearing on the Noncommunicating Children's Pain Checklist [24] is more likely indicative of fear or surprise, and “grinding teeth or clenching jaw,” also appearing on the Noncommunicating Children's Pain Checklist [24], is contrary to empirical findings that the mouth typically opens to varying degrees. Finally, some items seem to contradict other scale items, despite supposedly still indicating pain: for example, on the Derbyshire Children's Hospital Pain Tool [37], the pain indicator “quiet” in facial expression suggests mild pain, whereas “thrashing” in body movement suggests moderate pain. When items have been supported by empirical observation, such as “furrowing brow” on the Noncommunicating Children's Pain Checklist [24], “eyes squeezed” on the Checklist Pain Behavior [42], “deepened nasolabial furrow” also on the Checklist Pain Behavior [42], and “open mouth with lips pulled back at corners” on the Toddler Preschooler Postoperative Pain Scale [41], behavioral scales lead to higher levels of reliability and validity [53]. In particular, facial expression has attracted considerable attention as a sensitive and specific measure of pain. Other behavioral actions warrant further validation as reliable indicators of pain in clinical settings.

We note that on some scales certain cues function on their own, whereas on other scales these same cues are subcategories of other cues. For example, the Dalhousie Everyday Pain Scale [29] category for “protective behaviors” includes “reduction of activity” as an isolated cue, whereas the Behavioral Observation Tool places the same cue as a subcategory of “Body Movement” [32].

Weights assigned to the different pain behavior items may be problematic. Scales that add up the number of endorsed items to provide an index of pain severity de facto assign equivalent weights to items. It would seem that items that are only sensitive but not specific to pain (e.g., manifestations of aversive, but nonnoxious states) should receive less weight than both specific and sensitive items. Scales that include self-report items may artificially lower scores in children with cognitive or communication impairment if weight is attached to these items.

### 4.2. Automatic and Controlled Behaviors

The scales provide a mixture of automatic and controlled items, with other items ambiguous with respect to these categories. Research participants consistently identified variations in these qualities in the behavioral cues. Automatic items are perceived to be more reflexive, unconscious, nondeliberate, and stimulus driven, whereas controlled items are construed as reflecting conscious experience, deliberation, and effort to achieve some specific purpose [54]. Automatic behaviors would be seen as more clearly having a basis in somatic nociceptive or neuropathic processes (including central hypersensitivity and neuroplasticity), that is, reflecting pain, whereas controlled expression could have more diverse causes, including the foregoing as well as a potential nonnociceptive basis, such as an attempt to control the situation.

Most facial expressions were perceived to be automatic, which is consistent with the facial expression literature [55]. Furthermore, children are less able to control and suppress facial and body cues associated with deception [56, 57]. Paralinguistic vocalizations were also characterized as automatic, apparently due to features of stress in intonation, breathing, and speech qualities. These nociceptive reflexes have a protective function, either through homeostatic mechanisms or by attracting the attention of others and are considered automatic reactions [58]. A few motor cues resembling withdrawal reflexes were also rated as automatic, similar to recent findings [17].

The controlled behaviors were mostly comprised of verbalizations and instrumental motor activities. In this case, the motor behavior was distinct from the automatic reflexive behaviors, as it was characterized by extended duration and coordinated action [17]. The presence of verbalizations in this category was consistent with linguistic literature, which indicates that a high level of coordination and synchronization is required in the use of speech [59]. Some social behaviors were also identified as controlled, for example, “taking medication when he/she normally refuses” and “seeking physical comfort.”

### 4.3. Ambiguous Behaviors

Twenty-five cues were rated as ambiguous, indicating that the source of action is difficult to discern. In some cases, it was unclear whether the cue referred to a social behavior or motor activity. For example, “reduction of activity” was rated as ambiguous, possibly due to the lack of context. As a result, the cue could be seen as either a reflexive or controlled behavior. In any given scenario, context is important in drawing conclusions. In a clinical setting this might be more readily resolved.

Less commonly observed cues would appear to have greater corresponding ambiguity ratings. Certain cues, such as “eating less than usual” and “playing less than usual,” are not commonly found in pain scales and are not normally used as direct indicators of pain. Similarly, most facial cues were identified as automatic, although some, for example, “puckering” and “chewing” were found to be ambiguous. These cues tend not to be reported in the descriptive empirical literature on children's pain facial expressions and are also not prevalent in pain scales. Some cues were inherently unclear, such as “rear up body of the trunk/sit up.” Cultural backgrounds also need to be considered when explaining variations in interpretation of items, given research indicating that attitudes, beliefs, and psychological states of ethnic groups can affect the observational judgment of pain [60].

The substantial number and diversity of behaviors nominated as expressive of children's pain should not be surprising given the salience and potency of painful experiences: mobilization of biobehavioral resources in all their complexity would be anticipated. What perhaps is more important in scale development is selective refinement in identifying those cues that are the most sensitive and specific to pain. Items referring to pain specific behaviors should be favored. The behaviors listed provide numerous illustrations of actions likely to be sensitive, but not specific to pain, such as “crying.” These play an important role as they attract attention to the child and precipitate a search for more explicit information concerning the nature of the child's distress, for example, pain versus fear, anger, or irritability.

There is also merit in pursuing an understanding of the underlying structure of pain expression, with the functional value of the actions of particular importance. The functional distinction between protective and communicative actions has already been described. Nociceptive reflexes, guarded behaviors, or protective postures, for example, are useful sources of information about pain, but their communicative value would appear to be secondary to their functional value in avoiding or diminishing painful experience. This paper confirms that the dual neuroregulatory processes associated with automatic and controlled actions provide implicit structure for observer reactions to children's behavioral cues of pain. Automatic/reflexive actions are more likely to carry specific information concerning pain in fulfilling protective and communicative functions without the use of conscious deliberation, whereas controlled reactions represent efforts to cope with painful events, either through protective behavior or social communication requiring executive functions or higher levels of neuroregulatory systems [1]. This distinction would be expected to influence observer appraisals of the nature of the child's distress and have an impact on subsequent care or other dispositions when interacting with children.

## 5. Future Directions and Limitations

The present study is not without limitations. The country of residence for the majority of the sample recruited on Mechanical Turk was India (71.9%). Although previous studies using Mechanical Turk estimated that 70–80% of registered users live in the United States and provide a more representative sample of Americans than traditional undergraduate student samples [61, 62], the demographic characteristics of individuals using Mechanical Turk have changed in the last five years. More recently, more people using Mechanical Turk are from India [63], a finding that is reflected in the sample composition in the present study. It is possible that the cultural beliefs, practices, and biases of participants may have influenced how participants rated the automaticity of various pain behaviors in a way that may overrepresent cultural differences from one country more than individuals from other parts of the world. Further research is needed to determine whether the same factor structure would be found in different populations.

Furthermore, participants were asked to picture a child between the ages of 4 and 12 displaying the list of pain behavior cues. It is possible that participants may have imagined the expression/display of pain differently, which may have influenced the subsequent ratings of automaticity for the behavior. Future research should consider using visual stimuli, such as pictures or videos of children displaying the various pain cues to determine if the pain cues would have the same underlying factor structure.

The present findings may help inform the development of a new observation pain scale to improve pain assessment in children. Although it is possible that items with higher factor loadings may be good candidates for inclusion on a new scale, it would be important to evaluate the validity and reliability of each pain cue to determine whether each item can discriminate between a child experiencing pain from a child not experiencing pain or another negative affective state. Although we suspect that some items will be more likely to have higher sensitivity and specificity than other items (e.g., cues that refer to specific behaviors, describing facial expressions), further clinical research is required to confidently make this distinction.

## Figures and Tables

**Table 1:** Final list of pain behaviors extracted from pediatric observational pain scales that loaded on factor 1, which we labeled as the “Controlled” factor.

Pain behavior	Factor 1	Factor 2	Factor 3	Factor 4
Taking medication when he/she normally refuses	.626	−.153	.064	.069
Seeking physical comfort or closeness	.613	−.008	−.098	.335
Pulling head away	.442	.222	.161	−.017
Lying very still	.601	−.068	.010	.280
Screaming	.492	.266	.115	−.027
Shifting torso	.484	.081	.283	.047
Making aggressive physical contact	.738	.156	.014	.206
Resisting being moved	.701	−.097	.121	.180
Cursing	.694	.006	.087	−.055
Raising voice	.689	.210	.072	.046
Asking for help	.689	−.104	−.094	.083
Complaining of pain	.548	.009	−.045	.168
Self-harming	.486	−.156	.045	−.046
Angry verbalizations	.650	−.066	.203	−.014
Whining	.617	.069	.274	−.099
Quiet	.490	−.037	.202	.182
Kicking	.692	−.016	.218	−.114
Resistant	.604	−.119	.292	.091

**Table 2:** Final list of pain behaviors extracted from pediatric observational pain scales that loaded on factor 2, which we labeled as the “Automatic” factor.

Pain behavior	Factor 1	Factor 2	Factor 3	Factor 4
Socially withdrawn	.181	.382	.050	.172
Wincing	−.241	.555	.087	.146
Deepening of nasolabial furrow	−.222	.539	.140	.048
Whimpering	.164	.531	.022	.142
Flailing arms and legs	.323	.476	.067	.197
Grimacing	.082	.611	−.013	−.004
Hard to console or comfort	.190	.539	.072	.204
Watery eyes	−.272	.602	.172	−.105
Tensing up	−.122	.674	−.029	.066
Restless	−.029	.536	.151	.040
Squeezing eyes	.150	.370	.184	.017
Grinding teeth	−.321	.576	.158	.072
Widening eyes	−.106	.496	.318	−.109
Hyper alert	.011	.432	.229	.261
Irregular, indrawn breathing	−.423	.544	.228	.220
Crying	.288	.403	.292	−.134
Tears running down face	−.099	.553	.173	−.047
Squinting	−.154	.498	.148	−.276
Moaning	.072	.403	.281	.167
Shivering torso	−.502	.496	.273	.096
Looking pale	−.550	.511	.191	.214
Gasping	−.455	.474	.295	−.074
Has disturbed sleep	−.545	.482	.249	.169

**Table 3:** Final list of pain behaviors extracted from pediatric observational pain scales that loaded on factor 3 and/or factor 4, loaded on two or more factors, or did not load on any factors. This factor was labeled as the “Ambiguous” factor.

Pain behavior	Factor 1	Factor 2	Factor 3	Factor 4
Reduction of activity	.244	.157	.120	.410
Puckering	.134	.200	.361	.046
Rear up body of the trunk/sit up	.474	−.052	.372	.105
Jerking	−.094	.323	.478	.154
Furrowing brow	.223	.444	.377	−.103
Opening mouth	.272	.354	.429	−.201
Eating less than usual	.113	.157	.263	.440
Flexion or tightening of toes	−.140	.476	.470	−.016
Localizing pain	.136	.163	.173	.064
Arching back	.004	.274	.443	.203
Thrusting tongue out	.379	−.003	.529	.033
Rubbing	.371	.124	.398	.070
Grunting	.069	.345	.475	.082
Chewing	.252	.042	.490	.031
Looking more flushed than normal	−.566	.470	.338	.108
Irritable	.113	.259	.518	.154
Corners of mouth downward	−.017	.348	.548	−.109
Eyes almost closed	−.045	.246	.393	.151
Being difficult to distract	.272	.012	.394	.262
Playing less than usual	.152	.158	.390	.481
Drawn up with arms and legs to the body	.364	.173	.290	.167
Flexion or tightening of fingers	−.009	.384	.295	.038
Clenching jaw	−.024	.451	.315	−.039
Protecting/favoring/guarding part of body that hurts	.201	.330	.155	.044
Squirming	.217	.324	.247	.030
